# Tailoring deep brain stimulation for sleep: using actigraphy to understand the relationship between Parkinsonian brain activity and behavioral state

**DOI:** 10.1093/sleep/zsaf066

**Published:** 2025-03-15

**Authors:** Joram J van Rheede, Andrew Sharott

**Affiliations:** MRC Brain Network Dynamics Unit, Nuffield Department of Clinical Neurosciences, University of Oxford, Oxford, UK; Institute of Biomedical Engineering, Department of Engineering Science, University of Oxford, Oxford, UK; MRC Brain Network Dynamics Unit, Nuffield Department of Clinical Neurosciences, University of Oxford, Oxford, UK

Deep brain stimulation (DBS) is a bioelectronic therapy for neurological disorders. It acts through electrical stimulation of a target brain structure that is part of a dysfunctional network and has been particularly successful in the treatment of Parkinson’s disease (PD) [1]. In addition to motor symptoms, PD is characterized by debilitating and multifaceted sleep problems ranging from insomnia and sleep fragmentation to REM sleep behavior disorder [2, 3]. It is therefore important that DBS does not exacerbate such sleep problems. Research into the effects of long-term continuous subthalamic nucleus (STN) DBS for PD on patients’ sleep has generally found an overall beneficial effect of DBS [4, 5]. However, the interaction between DBS and sleep is still incompletely understood, and DBS may provide opportunities to target brain activities that cause sleep disturbance more proactively.

While the exact mechanisms of DBS’s therapeutic effect remain a matter of active investigation, it is becoming clear that it acts through a frequency- and amplitude-dependent modulation of the targeted brain network [6]. In PD, the most common approach is to use high-frequency (~130 Hz) stimulation of the STN or the globus pallidus. DBS parameters for PD are set during a programming session with a clinician, and the therapy runs continuously with the same stimulation parameters until a next clinical visit. However, this requires a careful balance between therapeutic efficacy and side effects, and patients can still experience daily periods of suboptimal treatment. A key research area, therefore, has been the development of systems that can adapt stimulation to therapeutic need autonomously and in real time (*closed-loop* or *adaptive* DBS) [7]. Hitherto, these approaches have mostly used real-time tracking of the amplitude of beta-frequency oscillations (~13–30 Hz) in the basal ganglia local field potential (LFP), a well-established electrophysiological marker of akinetic/rigid symptoms [8–13], to control the timing or amplitude of stimulation. Adaptive DBS has led to improvements over continuous DBS in laboratory settings [12, 14] and is now being tested in the large-scale ADAPT trial [15].

In addition to their relationship to motor symptoms in PD, laboratory studies with patients with externalized leads have shown that the amplitude of beta oscillations is strongly affected by sleep [16, 17]. However, to fully understand how such fluctuations are affected by therapeutic stimulation and daily activities and rhythms, longitudinal measurements are required. With the advent newer DBS devices [18], it has become possible to monitor neural oscillatory activity over multiple days or weeks during active high-frequency DBS therapy. Recent studies have exploited this capacity and shown that beta oscillations exhibit consistent diurnal fluctuations in amplitude, with beta activity being generally reduced at night when patients are assumed to be asleep [19, 20]. However, these studies employed a retrospective data analysis approach and did not prospectively include independent measures of behavioral state.

In the current issue of SLEEP, Baumgartner and colleagues use actigraphy alongside on-device long-term intracranial monitoring of STN LFPs in a cohort of PD patients [21]. They show consistent fluctuation of beta (and high alpha) STN LFP power associated with changes in sleep or wakefulness state as determined by actigraphy, with higher power in the target bands during wakefulness compared with sleep. This confirms that it is indeed transitions in behavioral state that drive transitions between epochs of low and high beta power. From their comparison of the distributions of beta during sleep and waking epochs, it appears that beta power at any given moment is highly predictive of behavioral state. The authors’ observation that the sleep/wake differences in beta do not change according to disease or DBS duration suggests that these changes with behavioral state are a very stable property of cortico-basal-ganglia circuits in PD. Indeed, even a within-patient change in stimulation amplitude, though reducing overall beta power, had limited effect on the diurnal variations. A lack of relationship between beta peak frequency and diurnal pattern contrasts with another recent study [20]—though the current study was not specifically powered for this comparison.

A potential caveat is that, while actigraphy is a logical starting point for determining behavioral or vigilance state as devices are readily available and are a low burden for research participants and patients, its use for sleep classification has limitations. Validation studies tend to focus on broader classifications than used in traditional sleep scoring [22], and there are questions about using metrics validated in the general population on patient populations (particularly patients with movement disorders). To gain further insights into how beta power fluctuates with specific sleep stages during active DBS over longer time scales in older patients, additional measures such as portable or in-ear EEG could be considered [23–25]. Nevertheless, taken together with previous work [16, 17, 26–28], the drop in beta associated with sleep appears to be a robust finding.

For the delivery of closed-loop DBS, control algorithms need to be resilient to changes that affect the brain on multiple timescales, including the sleep-related beta changes observed here [29–31]. Current adaptive DBS strategies, such as that used in the ADAPT trial, are aimed at controlling beta oscillations during the day, with the goal of suppressing symptom-related activity as it arises. The sleep-related drop in beta would see such an algorithm turn down therapy—but is this the best action to take? In fact, a set of recent studies make it clear that despite its overall lower amplitude, beta activity during sleep is mechanistically involved in sleep disruption [26–28, 32]. The clinically relevant dynamic range of beta activity may therefore be different during sleep, and algorithm thresholds may need adjustment. Care must also be taken if a patient’s beta peak overlaps with the natural range of healthy sleep spindle activity [33–35].

Delivery of sleep-aware DBS requires on-device detection of sleep to trigger a change in stimulation algorithm. While this has been trialed in research-only high-bandwidth devices enabling signal processing off-device [36, 37], devices such as the Medtronic Percept are only able to continuously monitor an individual power band. The featured study adds to other recent evidence that it may be possible to track sleep from these less complex neural signatures aleady available to devices used in clinical practice [38, 39]. The stable and predictive nature of beta power could make it a suitable input to an on-device brain-state classifier for a future sleep-aware DBS system. Other clear candidates are bands associated with specific stages of sleep such as slow, delta, and spindle ranges [40, 41]. Additionally, the demonstrated link between actigraphy data, behavioral state, and Parkinsonian beta behavior could be exploited by future DBS platforms that have access to built-in accelerometers [42, 43].

Tailoring DBS to sleep offers an exciting new avenue for the field of DBS and neuromodulation. This is also relevant beyond its application in PD—for instance, it is well established that in epilepsy, seizures can be strongly linked to sleep and circadian rhythm [44, 45], but also that neuromodulation therapy might interfere with sleep [46, 47], requiring a careful consideration of the optimal therapy across the sleep–wake cycle. Moreover, it is increasingly clear that there is a bidirectional relationship between neurodegenerative disorders such as PD and sleep and circadian rhythm disruption [48–50]. If DBS can target this disruption and improve patient sleep, it has a real opportunity to move beyond controlling symptoms and become a disease-modifying therapy.
